# Nossos Pacientes Merecem Mais

**DOI:** 10.36660/abc.20230626

**Published:** 2023-10-03

**Authors:** Roberto Muniz Ferreira

**Affiliations:** 1 Universidade Federal do Rio de Janeiro Instituto do Coração Edson Saad Rio de Janeiro RJ Brasil Universidade Federal do Rio de Janeiro, Instituto do Coração Edson Saad, Rio de Janeiro, RJ – Brasil; 2 Hospital Samaritano Botafogo RJ Brasil Hospital Samaritano, Botafogo, RJ – Brasil

**Keywords:** Betacoronavirus, Pandemia, COVID-19, Infecções por Coronavirus, Hidroxicloroquina, Azitromicina, Medicina Baseada em Evidências

A tomada de decisão clínica implica que múltiplas opções são possíveis para qualquer situação médica. Felizmente, a ciência está em constante evolução e continuará a fazê-lo de forma incremental. Assim, os médicos devem analisar rotineiramente resultados anteriores de circunstâncias semelhantes para aumentar a probabilidade de selecionar a alternativa mais apropriada. Discernir os dados que podem ajudar na tomada de decisões a partir de informações científicas inconsistentes é um requisito fundamental da medicina baseada em evidências (MBE).

Há mais de 40 anos, a MBE tem sido a pedra angular da prática clínica, melhorando a eficácia e a segurança envolvidas nas decisões médicas, de acordo com o conhecimento científico mais atual.^1^ No entanto, as publicações nem sempre aderem a conceitos metodológicos validados e podem basear-se apenas em motivações não científicas. Quando as crenças pessoais ofuscam a ciência médica, isso ocorre invariavelmente em detrimento do cuidado ao paciente.^2^ Por outro lado, a MBE fornece um método sistemático e organizado para avaliar informações científicas em diversas formas de literatura médica.

Gerir a incerteza é um dos principais desafios da prática médica, especialmente em situações sem precedentes. A ciência tem limitações e mesmo uma investigação extensa pode não eliminar a incerteza. Mais importante ainda, lidar com a incerteza é crucial para evitar que os pacientes sejam expostos a respostas potencialmente inadequadas por parte dos médicos.^3^ Reconhecer quando aceitar a espera vigilante e não adotar impulsivamente tratamentos não comprovados é vital para evitar que as intervenções se tornem um perigo maior do que a própria doença.

A pandemia da Doença do Coronavírus 2019 (COVID-19) revelou muitas inseguranças relacionadas à MBE na comunidade médica. Num cenário de desespero e insegurança, os profissionais de saúde difundiram vários medicamentos não comprovados e potencialmente prejudiciais como tratamentos eficazes. Vários médicos enfrentaram a incerteza ao aventurarem-se numa abordagem de “tentativa e erro”, acreditando que tais circunstâncias justificavam qualquer conduta médica, independentemente dos riscos potenciais.^4^ Neste contexto, a hidroxicloroquina/cloroquina (HCQ/CQ), a ivermectina e a azitromicina (AZT) foram massivamente empregados como tratamentos supostamente eficazes. Notavelmente, mesmo depois de várias publicações terem sugerido os potenciais danos destas drogas, muitos confiaram em crenças pessoais e coletivas infundadas como pilares da tomada de decisões.^5-8^

O artigo publicado nesta edição dos Arquivos Brasileiros de Cardiologia fornece mais informações sobre as consequências das tomadas de decisão sem suporte que ocorreram durante a pandemia. Os autores apresentaram dados retrospectivos de 673 pacientes hospitalizados com COVID-19 no Brasil tratados com HCQ/CQ, com ou sem AZT, entre março e setembro de 2020. O estudo fez parte do Registro Brasileiro de COVID-19, uma iniciativa multicêntrica que inscreveu consecutivamente pacientes com doença confirmada laboratorialmente.^9^ O objetivo foi comparar os resultados clínicos e eletrocardiográficos intra-hospitalares com controles adequadamente pareados. Ao longo do período de estudo de 6 meses, mais de 145 mil mortes por COVID-19 foram registradas no Brasil.^10^

Os pacientes eram em sua maioria mulheres (55,9%), com idade média de 58 anos. A hipertensão estava presente em 49% e aproximadamente 30% eram diabéticos. Diferentes regimes de hidroxicloroquina foram administrados a 90% dos pacientes, enquanto 9,9% e 88,1% foram tratados com cloroquina e AZT, respectivamente. Um eletrocardiograma (ECG) estava disponível em apenas 42% dos pacientes internados, embora 60% tivessem doença cardiovascular. Não houve diferenças significativas na prevalência de achados basais anormais no ECG em comparação com o grupo controle.

O grupo tratado apresentou maior tempo de internação hospitalar (9,0 dias vs. 8,0 dias, p<0,001) e tendência à necessidade adicional de ventilação mecânica (27% vs. 22,3%, p=0,074), mesmo após exclusão daqueles que receberam apenas os medicamentos após a intubação. Novas alterações no ECG foram identificadas principalmente no mesmo grupo (13,2% vs. 8,2%, p=0,004), primariamente devido ao prolongamento do intervalo QTc (3,6% vs. 0,4%, p<0,001). Não houve diferenças na admissão em terapia intensiva, arritmias ventriculares ou mortalidade hospitalar entre os dois grupos (18,9% vs. 18,0%, p=0,682).

Embora os resultados refiram-se a uma era pré-vacinação, os autores devem ser elogiados por apresentarem um reflexo das perigosas vias de tratamento adotadas desde o início da pandemia de COVID-19. À primeira vista, a falta de uma incidência significativamente maior de resultados adversos em pacientes tratados com HCQ/CQ e AZT pode aparentemente fornecer garantias de que nenhum dano foi associado a estes medicamentos. No entanto, além das limitações dos dados retrospectivos, qualquer intervenção só deve ser implementada quando a segurança e a eficácia tiverem sido consistentemente demonstradas através dos requisitos apropriados da MBE. O presente estudo fornece evidências de dados da vida real de que estes pré-requisitos não foram alcançados para HCQ/CQ e AZT na COVID-19.

Três anos depois, as consequências de desconsiderar a ciência numa crise de saúde tão devastadora tornaram-se cada vez mais evidentes. Quando os recursos são escassos, as despesas desnecessárias com intervenções ineficazes e possivelmente perigosas não devem ser ignoradas ou esquecidas. Devemos questionar o que poderia ter sido realizado e quantas vidas poderiam ter sido salvas de outra forma. Como médicos, apenas acreditar que algo foi aprendido com os erros anteriores não é suficiente. É obrigatória uma reflexão profunda sobre o que ocorreu e como melhorar para as gerações futuras. Nossos pacientes merecem mais.
