# Supplementary material for: Nossos Pacientes Merecem Mais
Source: Arq Bras Cardiol. 2023 Oct 3;120(9):e20230626. [Article in Portuguese] doi: 10.36660/abc.20230626 (PMC10547431; doi:10.36660/abc.20230626)
Supplement: Translation

# Translation: Our Patients Deserve More

**Authors:** Roberto Muniz Ferreira

**Affiliations:** 1 Brasil Universidade Federal do Rio de Janeiro, Instituto do Coração Edson Saad, Rio de Janeiro, RJ – Brasil; 2 Brasil Hospital Samaritano, Botafogo, RJ – Brasil

**Keywords:** Betacoronavirus, Pandemics, COVID-19, Coronavirus Infections, Hydroxychloroquine, Azithromycine, Evidence-Based Medicine

Clinical decision-making implies that multiple options are possible for any given medical situation. Fortunately, science is constantly evolving and will continue to do so incrementally. Thus, physicians should routinely analyze previous outcomes of similar circumstances to increase the probability of selecting the most appropriate alternative. Discerning data that may assist in decision-making from inconsistent scientific information is a fundamental requirement of evidence-based medicine (EBM).

For over 40 years, EBM has been the cornerstone of clinical practice, improving the efficacy and safety involved with medical decisions, according to the most current scientific knowledge.^1^ Nevertheless, publications do not always adhere to validated methodological concepts and may rely solely on non-scientific motivations. When personal beliefs overshadow medical science, it invariably occurs to the detriment of patient care.^2^ Conversely, EBM provides a systematic and organized method to appraise scientific information in various medical literature forms.

Managing uncertainty is one of the main challenges of medical practice, especially in unprecedented situations. Science has limitations, and even extensive research may not eliminate uncertainty. Most importantly, coping with uncertainty is crucial to prevent patients from becoming exposed to potentially maladaptive responses by physicians.^3^ Recognizing when to accept watchful waiting and not impulsively adopt unproven treatments is vital to preclude interventions from becoming a greater hazard than the disease itself.

The Coronavirus Disease 2019 (COVID-19) pandemic uncovered many EBM-related insecurities within the medical community. In a scenario of despair and insecurity, healthcare professionals disseminated various unproven and potentially detrimental medications as efficacious treatments. Several physicians confronted uncertainty by venturing into a “trial and error” approach, believing that such circumstances justified any medical conduct, regardless of potential risks.^4^ In this context, hydroxychloroquine/chloroquine (HCQ/CQ), ivermectin, and azithromycin (AZT) were massively employed as allegedly effective treatments. Remarkably, even after various publications suggested the potential harms of these drugs, many relied on unfounded personal and collective beliefs as the pillars of decision-making.^5-8^

The article published in this Arquivos Brasileiros de Cardiologia issue provides further insight into the consequences of unsupported decision-making that transpired during the pandemic. The authors presented retrospective data on 673 hospitalized patients with COVID-19 in Brazil treated with HCQ/CQ, with or without AZT, between March and September 2020. The study was part of the Brazilian COVID-19 Registry, a multicenter initiative that enrolled consecutive patients with laboratory-confirmed disease.^9^ The objective was to compare in-hospital clinical and electrocardiographic outcomes with appropriately matched controls. Throughout the 6 month study period, over 145 thousand COVID-19 deaths were registered in Brazil.^10^

Patients were mostly female (55.9%), with an average age of 58. Hypertension was present in 49%, and approximately 30% were diabetic. Different regimens of hydroxychloroquine were administered to 90% of patients, whereas 9.9% and 88.1% were treated with chloroquine and AZT, respectively. An electrocardiogram (ECG) was available in only 42% of admitted patients, although 60% knowingly had cardiovascular disease. There were no significant differences in the prevalence of abnormal ECG baseline findings compared to the control group.

The treated group experienced a greater length of hospital stay (9.0 days vs. 8.0 days, p<0.001) and a trend towards additional mechanical ventilation requirements (27% vs. 22.3%, p=0.074), even after excluding those who only received the drugs after intubation. Novel ECG abnormalities were mostly identified in the same group (13.2% vs. 8.2%, p=0.004), mainly due to QTc prolongation (3.6% vs. 0,4%, p<0.001). There were no differences in intensive care admission, ventricular arrhythmias, or in-hospital mortality between both groups (18.9% vs. 18.0%, p=0.682).

Although the results relate to a pre-vaccination era, the authors should be commended for presenting a portrayal of the hazardous treatment pathways adopted since the beginning of the COVID-19 pandemic. At first glance, the lack of a significantly greater incidence of adverse outcomes in patients treated with HCQ/CQ and AZT may seemingly provide reassurance that no harm was associated with these medications. However, aside from the limitations of retrospective data, any intervention should only be implemented when safety and effectiveness have been consistently demonstrated through the appropriate requirements of EBM. The current study provides evidence from real-life data that these prerequisites were not achieved for HCQ/CQ and AZT in COVID-19.

Three years later, the consequences of disregarding science in such a devastating health crisis have become increasingly apparent. When resources are scarce, unnecessary expenditures with ineffective and possibly hazardous interventions should not be ignored or forgotten. We must question what could have been accomplished and how many lives may have been saved otherwise. As physicians, merely believing that something was learned from previous mistakes is not enough. A profound reflection on what occurred and how to improve for future generations is mandatory. Our patients deserve more.
